# Urinary ATP Levels Are Controlled by Nucleotidases Released from the Urothelium in a Regulated Manner

**DOI:** 10.3390/metabo13010030

**Published:** 2022-12-24

**Authors:** Alejandro Gutierrez Cruz, Mafalda S. L. Aresta Branco, Brian A. Perrino, Kenton M. Sanders, Violeta N. Mutafova-Yambolieva

**Affiliations:** Department of Physiology and Cell Biology, School of Medicine, University of Nevada Reno, Reno, NV 89557, USA

**Keywords:** ATP, urothelium, bladder, nucleotidases, purinergic, purine nucleotides, bladder lumen, bladder lamina propria, ATP hydrolysis, CD73

## Abstract

Adenosine 5′-triphosphate (ATP) is released in the bladder lumen during filling. Urothelial ATP is presumed to regulate bladder excitability. Urinary ATP is suggested as a urinary biomarker of bladder dysfunctions since ATP is increased in the urine of patients with overactive bladder, interstitial cystitis or bladder pain syndrome. Altered urinary ATP might also be associated with voiding dysfunctions linked to disease states associated with metabolic syndrome. Extracellular ATP levels are determined by ATP release and ATP hydrolysis by membrane-bound and soluble nucleotidases (s-NTDs). It is currently unknown whether s-NTDs regulate urinary ATP. Using etheno-ATP substrate and HPLC-FLD detection techniques, we found that s-NTDs are released in the lumen of ex vivo mouse detrusor-free bladders. Capillary immunoelectrophoresis by ProteinSimple Wes determined that intraluminal solutions (ILS) collected at the end of filling contain ENTPD3 > ENPP1 > ENPP3 ≥ ENTPD2 = NT5E = ALPL/TNAP. Activation of adenylyl cyclase with forskolin increased luminal s-NTDs release whereas the AC inhibitor SQ22536 had no effect. In contrast, forskolin reduced and SQ22536 increased s-NTDs release in the lamina propria. Adenosine enhanced s-NTDs release and accelerated ATP hydrolysis in ILS and lamina propria. Therefore, there is a regulated release of s-NTDs in the bladder lumen during filling. Aberrant release or functions of urothelial s-NTDs might cause elevated urinary ATP in conditions with abnormal bladder excitability.

## 1. Introduction

Adenosine 5′-triphosphate (ATP) is released in the bladder lumen in response to stretch or chemical stimulation [1,2,3,4]. Urothelial ATP is proposed to serve mechanotransduction by activating afferent nerve termini in the bladder mucosa to signal bladder fullness to the CNS [5,6], to control urothelial surface area as the bladder stretches during filling [7,8], to stimulate release of bioactive mediators in the bladder wall [9], and to possibly activate smooth muscle contraction directly [10]. ATP is released in the bladder lumen at both low and high filling volumes [3,11], suggesting that mechanosensitive transduction is not the only function of ATP in the bladder. The role of ATP at low filling volumes remains to be elucidated. In addition to its roles in physiological bladder functions, ATP likely contributes to bladder dysfunctions that are characterized with urinary urgency and increased voiding frequency, with or without urge incontinence. For example, ATP is increased in the urine in patients with overactive bladder (OAB) syndrome [11,12], interstitial cystitis and bladder pain syndrome [13,14,15], and bladder infection and inflammation [16,17,18]. Conversely, decreased intravesical ATP has been found in patients with refractory detrusor overactivity and bacteriuria [19] and in patients with underactive bladder syndrome [20]. Such observations have increasingly suggested ATP as a relevant biomarker of bladder dysfunctions [11,12,21,22,23,24]. It was recently alluded that ATP might be a better urinary marker than acetylcholine and prostaglandin E2, as the majority of ATP in urine is thought to originate in the lower urinary tract (LUT), whereas the other two substances may also arise from extraction from the plasma into the urine in the kidneys [25].

Voiding dysfunctions have been linked not only to diseases of the LUT, but also to systemic diseases such as diabetes [26,27,28,29], obesity [29,30], hyperlipidemia [31,32], and hypertension [33,34,35] or with metabolic syndrome that is defined by impaired glucose tolerance, central obesity, hypertension and dyslipidemia [36,37,38]. Metabolic syndrome and OAB may share common pathophysiology mechanisms [39] since patients with metabolic syndrome exhibit a higher incidence of OAB [40]. Impaired purinergic signaling in the bladder has been demonstrated in animal models of diabetes, dyslipidemia, obesity and hypertension [41,42,43,44] and, therefore, ATP and other purines might be involved in the development of voiding dysfunctions associated with systemic diseases, including the metabolic syndrome.

The amount of ATP that is available in the vicinity of P2 purine receptors in effector cells is a result of two processes that occur almost simultaneously in the extracellular space—release and metabolism of ATP. Many studies claim to measure extracellular *release* of ATP while, in fact, they likely measure the result of released minus removed ATP. The impact of ATP hydrolysis on availability of ATP at receptor sites or in body fluids (e.g., urine) might be substantial, but it has largely been understudied. Several lines of evidence suggest that significant ATP hydrolysis likely occurs in the bladder wall. First, immunohistochemistry and protein biochemistry studies have demonstrated the presence of several enzymes that hydrolyze ATP in the murine bladder urothelium [45,46]. Second, ATP and its metabolites adenosine 5′-diphosphate (ADP), 5′-monophosphate (AMP), and adenosine have been detected on both sides of the urothelium at the end of bladder filling [3,4]. Third, at the end of filling, ATP represented only ~5% and ~12% of the total purine pool in lamina propria and bladder lumen, respectively [4]. Finally, mechanosensitive hydrolysis of ATP and ADP in the bladder lamina propria has recently been demonstrated [47]. Taken together, such observations suggest that ATP hydrolysis likely plays a significant role in determining the effective concentrations of ATP in the bladder wall and in the urine. Furthermore, the asymmetrical bioavailability of ATP and its metabolites in lamina propria and bladder lumen suggests that the mechanisms of ATP degradation might not be identical in the luminal (i.e., mucosal) and abluminal (i.e., serosal) sides of the urothelium [4]. Therefore, observations made in the bladder lumen cannot be used as a surrogate for events in the opposite side of the urothelium [48].

Extracellular ATP is degraded sequentially to ADP, AMP, and adenosine by four families of enzymes that include ecto-nucleoside triphosphate diphosphohydrolases (ENTPDases), ecto-nucleotide pyrophosphatase/phosphodiesterases (ENPPs), alkaline phosphatase (ALPL)/tissue-nonspecific isozyme alkaline phosphatase (TNAP), and 5′-nucleotidase (NT5E/CD73) [49]. Membrane-bound nucleotidases are expressed in practically all cell types whereas soluble forms of nucleotidases may have more specialized location and functions [49,50] making them an attractive potential therapeutic target. For simplicity, we refer to membrane-bound (ecto-) nucleotidases and soluble nucleotidases as e-NTDs and s-NTDs throughout this study. It is conceivable to assume that s-NTDs are present in the urine, since investigators commonly freeze-dry urine samples to diminish the hydrolysis of ATP in urine. Soluble ATPases in the urine, if any, might originate from sources that are different from the bladder mucosa. For example, soluble nucleotidases are released in the renal tubules [51] or from bacteria in the urine [16,52]. Nucleotidases might also be released from the bladder mucosa, but this possibility has not been explored thoroughly. Therefore, the present study was designed to investigate the hypothesis that, in analogy to the bladder lamina propria [47], enzymes that hydrolyze ATP are released in the bladder lumen during filling and consequently regulate the urinary levels of ATP.

## 2. Methods

### 2.1. Animals

C57BL/6J and *Nt5e^−/−^* mice (Jackson Laboratory, Bar Harbor, MN, USA), 12–16 weeks post-partum, were euthanized by sedation with isoflurane (AErrane; Baxter, Deerfield, IL, USA) followed by cervical dislocation and exsanguination. The urinary bladder was removed and placed in oxygenated ice-cold Krebs-bicarbonate solution (KBS) for further dissection. KBS had the following composition (mM): 118.5 NaCl, 4.2 KCl, 1.2 MgCl_2_, 23.8 NaHCO_3_, 1.2 KH_2_PO_4_, 11.0 dextrose, and 1.8 CaCl_2_ (pH 7.4).

### 2.2. Ethical Approval

Animals were maintained and experiments were performed in accordance with the National Institutes of Health Guide for the Care and Use of Laboratory Animals and the Institutional Animal Use and Care Committee at the University of Nevada.

### 2.3. Denuded Bladder Preparation

The urinary bladder was dissected out after a midline laparotomy and placed in a dissecting dish containing oxygenated ice-cold KBS. Following the removal of fat and connective tissue, the bladder detrusor muscle was removed as described previously [4,47,53]. The bladder preparation was catheterized through the urethra with a 2 cm P20 tubing and placed in a 3 mL custom made water-jacketed chamber with a Sylgard bottom filled with KBS with constant O_2_ supply. The bladder catheter was connected to an infusion syringe pump (Genie Touch, Kent Scientific, Torrington, CT, USA) for bladder filling.

### 2.4. Evaluation of s-NTD Activities in the Lumen of Detrusor-Free Bladder Preparations

Following 30 min equilibration, the KBS in the chamber containing the bladder preparation was replaced with 3 mL of fresh KBS and the bladder was filled at 15 µL/min with oxygenated KBS to pre-voiding volume as previously described [4,47]. Then, the intraluminal solution (ILS) was transferred to a clean 600-µL Eppendorf tube. All reactions were carried out in 200 µL volumes at 37 °C. Substrate eATP or eAMP was added to the ILS to a final concentration of 2 µM. Following addition of substrate, 20 µL samples were collected from the reaction solution at 10 s, 2 min, 4 min, 6 min, 8 min, 10 min, 20 min, 30 min, 40 min and 60 min and diluted 10-fold with ice-cold citric phosphate buffer (pH 4.0) to stop the enzymatic reactions. Collected samples were compared with a 2 µM substrate in KBS that has not been in contact with enzymes. All samples were stored at −20 °C until final analysis. Substrate decrease and product increase was evaluated by HPLC-FLD methodology as described in Section 2.11. Each e-purine was expressed as percent of total purines [i.e., eATP + eADP + eAMP + e-adenosine (eADO) for eATP or eAMP + eADO for eAMP] detected in each sample. Only one substrate was used in each bladder preparation.

To determine whether ILS filtration would lead to loss of enzyme(s), ILS were first diluted to 2.9 mL with KBS and then they were concentrated using 4 mL Amicon Ultra centrifugal filter units with 10 kDa molecular weight cut-off (MWCO) pore size (Millipore Sigma, Burlington, MA, USA) by centrifugation at 4000× *g* for 25 min at 4 °C using swing bucket rotor (ThermoFisher Scientific SorvallST 40 R, Langenselbold, Germany) [47]. The concentrated ILS were brought to 200 µL with oxygenated KBS and enzymatic reactions were performed at 37 °C in an identical manner as the reactions in undiluted/unfiltered ILS.

### 2.5. Evaluation of Combined NTD Activities in the Lumen of Detrusor-Free Bladder Preparations

After removal of detrusor muscle, the bladder preparation was cannulated through the urethra with a double lumen catheter (CR-DL, Braintree Scientific, Inc., Braintree, MA, USA) and was placed in a 3 mL chamber filled with oxygenated KBS as described in Section 2.4. Following equilibration for 30 min, the bladder preparation was filled with KBS containing 2 µM eATP at 15 µL/min to pre-voiding volume through one of the two lines of the double lumen catheter. Then, the infusion was stopped and 20-µL samples from the IL solution were withdrawn through the second line of the catheter using a 50-µL Hamilton syringe (Hamilton Company, Inc., Reno, NV, USA) at 10 s, 2 min, 4 min, 6 min, 8 min, 10 min, 20 min, 30 min, 40 min and 60 min and placed in HPLC inserts filled with 180 µL cold citric buffer to stop the enzymatic reactions. The samples were processed further for detection of e-purines as described in Section 2.4.

### 2.6. Evaluation of s-NTDs Activities in the Lamina Propria of Detrusor-Free Bladder Preparations

In experiments designed to compare substrate degradation by soluble enzymes released simultaneously in the lamina propria and in the bladder lumen, 2.9 mL of bath solution that was in contact with the lamina propria during filling (termed extraluminal solution, ELS) was concentrated using 4 mL Amicon Ultra centrifugal filter units with a 10 kDa MWCO pore size by centrifugation at 4000× *g* for 25 min at 4 °C as described previously [47] and in Section 2.4. The concentrated ELS samples were brought to 200 µL with oxygenated KBS and enzymatic reactions were performed at 37 °C in an identical manner as the reactions in ILS.

### 2.7. Effects of Common ENTPDase Inhibitors on Soluble Nucleotidase Activities

To assess the effects of nucleotidase inhibitors, the bladder preparations were incubated with each inhibitor throughout dissection, equilibration, bladder filling and during the time-course reactions. The enzymatic reactions were conducted as described in Section 2.4. The following nucleotidase inhibitors were used: ARL67156 (100 µM in KBS, non-specific ENTPDase inhibitor), POM-1 (100 µM in KBS, non-specific ENTPDase inhibitor), ENPP1-Inhibitor-C (50 µM in DMSO 0.1%, ENPP1 inhibitor), PSB06126 (10 µM in DMSO 0.1%, ENTPD3 inhibitor), levamisole (1 mM in KBS, ALPL/TNAP inhibitor), and (−)-*p*-bromotetramisole oxalate (L-*p*-BT) (100 µM in DMSO 0.1%, ALPL/TNAP inhibitor). The effects of each inhibitor were compared with the effects of the corresponding vehicle. Only one inhibitor was tested in each bladder preparation.

### 2.8. Effects of Activation and Inhibition of Adenylyl Cyclases on the Release of s-NTDs in the Bladder Lumen and in Lamina Propria

To assess the effects of adenylyl cyclase (AC) activation, the bladder preparation was filled with forskolin (FSK, 10 µM or 25 µM) dissolved in DMSO 0.1% in KBS. FSK was also added to the bath with a nondistended (empty) bladder or during bladder filling (distended bladder). The ILS was collected at the end of bladder filling and placed in a 600-µL reaction tube. Samples of ELS (2.9 mL) bathing nondistended or distended preparations were concentrated as described in Section 2.6. and placed in 600-µL reaction tubes. eATP substrate was added to all reaction tubes and the reactions were performed as described in Section 2.4. To assess the effects of AC inhibition, the bladder preparation was incubated with the AC inhibitor SQ22536 (100 µM, dissolved in DMSO 0.2%) throughout dissection, equilibration, bladder filling and during the time-course reactions. The enzymatic reactions in ILS and concentrated ELS were performed as described for the experiments in the presence of FSK.

### 2.9. Effects of Adenosine on the Release of s-NTDs in the Bladder Lumen and the Lamina Propria

Following equilibration, the KBS in the chamber was replaced with fresh KBS containing 10 µM or 100 µM adenosine and the bladder was either left nondistended for the time equivalent to filling or it was filled (distended) at 15 µL/min with KBS containing adenosine to pre-voiding filling volume. This step ensured that soluble enzymes were released in both ILS and ELS in the presence of adenosine. To avoid interference of adenosine with the HPLC assay, the enzymatic reactions had to be performed in solutions that contained released enzymes but no adenosine. Therefore, 2.9 mL ELS and 2.9 mL ILS diluted in KBS were placed in centrifugal filter units and concentrated to approximately 80 µL as described in Section 2.6. In the next step, 2.9 mL of KBS was added to the centrifugal filter units with concentrated ELS or concentrated ILS and the samples were centrifuged again at 4000× *g* for 15 min. To ensure complete replacement of the solution containing adenosine with regular KBS, another 2.9 mL of KBS was added to the centrifugal units and concentrated at 4000× *g* for 25 min. The concentrated ELS and ILS solutions containing released enzymes were brought up to 200 µL with KBS and eATP substrate was added. Time-courses of enzymatic reactions following addition of eATP to reaction solutions were performed as described in Section 2.4. The hydrolysis of eATP in concentrated ILS and ELS containing enzymes released in the presence of adenosine was compared with eATP hydrolysis in regular KBS processed in the same manner as concentrated ILS and ELS samples.

### 2.10. Preparation of 1,N^6^-Etheno-Nucleotides

1,*N*^6^-etheno-ATP (eATP) and 1,*N*^6^-etheno-AMP (eAMP) were prepared as described previously [47]. Briefly, 0.2 mM ATP or AMP (dissolved in double distilled water) was acidified to pH 4.0 with citrate phosphate buffer. 2-Chloroacetaldehyde (1 M) was added and substrates were heated to 80 °C for 40 min to form 1,*N*^6^-ethenoderivatives of ATP and AMP, namely eATP and eAMP [54,55]. Substrates were further diluted in the reaction solution to 2 µM.

### 2.11. HPLC Analysis of 1,N^6^-Etheno-Nucleotides

A reverse phased gradient Agilent 1200 liquid chromatography system equipped with a fluorescence detector (FLD) (Agilent Technologies, Wilmington, DE, USA) was used to detect 1,*N*^6^-etheno-purines as described previously [3,4,47]. 1,*N*^6^-etheno-derivatized purines were detected by fluorescence at an excitation wavelength of 230 nm and emission wavelength of 420 nm [55]. ChemStation (v. B04-03) software (Agilent Technologies) was used to analyze areas under the peaks. Amounts of eATP, eADP, eAMP, and eADO were compared with 1,*N*^6^-etheno-derivatized purine standards (0.05–5 pmol).

### 2.12. Automated Capillary Electrophoresis and Immunodetection with Wes Simple Western

Capillary electrophoresis and Western blotting by Wes (Protein Simple, San Jose, CA, USA) were used to detect NTDs in ILS and in urothelium homogenates as described [47,56,57], ILS samples were collected at the end of bladder filling with KBS at 15 µL/min. Urothelium tissues from detrusor-free bladder preparations were homogenized in ice-cold lysis buffer (mM: 50 Tris-HCl pH 8.0, 60 β-glycerophosphate, 100 NaF, 2 EGTA, 25 sodium pyrophosphate, 1 DTT, 0.5% NP-40, 0.2% sodium dodecyl sulfate and protease inhibitors). Tissues were homogenized in 0.2 mL lysis buffer in a Bullet Blender (0.01% anti-foam C, one stainless steel bead per tube, speed 6, 5 min), then centrifuged at 16,000× *g*, for 10 min at 4 °C. Both ILS and urothelium homogenate supernatants were snap-frozen in liquid N_2_, and stored at − 80 °C for subsequent Wes analysis. Protein levels were analyzed using a Wes Simple Western instrument according to the Wes User Guide from ProteinSimple. The samples were mixed with the fluorescent 5× master mix (ProteinSimple) and then heated at 95 °C for 5 min. Boiled samples, biotinylated protein ladder, blocking buffer, primary antibodies, ProteinSimple horseradish peroxidase-conjugated anti-rabbit or anti-mouse secondary antibodies, luminol-peroxide and wash buffer were loaded into the Wes plate (Wes 12–230 kDa Pre-filled Plates with Split Buffer, ProteinSimple). The plates and capillary cartridges were loaded into the Wes instrument, and protein separation, antibody incubation and imaging were performed using default parameters. Compass software (ProteinSimple) was used to acquire the data, and to generate the virtual blot image reconstruction and chemiluminescence signal intensity electropherograms. The electropherogram shows the intensity detected along the length of the capillaries, and shows automatically detected peaks, that can be quantified by calculation of the area under the curve (AUC). Protein levels are expressed as the AUC of the peak chemiluminescence intensity.

### 2.13. Antibodies

The following primary antibodies were used for Wes analysis: from Cell Signaling Technology, rabbit anti-ENTPD1, (E2X6B); from ThermoFisher, sheep anti-ENTPD2, (PA5-47777); rabbit anti-ENTPD3, (PA5-87888); rabbit anti-ENPP1, (PA5-17097); rabbit anti-ENPP3, (PA5-67955); from EpiGentek, rabbit anti-ENTPD8, (A62482); from Abcam, rabbit anti-NT5C1A, (ab190214); and, from ABclonal, rabbit anti-CD73/NT5E, (A2029) and rabbit anti ALPL/TNAP, (A1080). The antibodies were either knockout validated by the vendor or validated in mouse brain homogenates as described [47].

### 2.14. Drugs and Reagents

Adenosine, ATP, ADP, AMP, dimethyl sulfoxide (DMSO) (Sigma-Aldrich, St. Louis, MO, USA), 6-[(3-aminophenyl)methyl]-N,N,5-trimethyl-[1,2,4]triazolo[1,5-a]pyrimidin-7-amine (ENPP1 Inhibitor C) (Cayman Chemicals, Ann Arbor, MI, USA), (−)-*p*-bromotetramisole oxalate (L-*p*-BT) (MedChemExpress, Monmouth Junction, NJ, USA); 6-*N*,*N*-Diethyl-D-β,γ-dibromomethyleneATP trisodium salt (ARL67156), sodium metatungstate (POM-1), and 1-Amino-4-(1-naphthyl)aminoanthraquinone-2-sulfonic acid sodium salt (PSB06126), FSK, 9-(Tetrahydro-2-furanyl)-9*H*-purin-6-amine (SQ22536) (Bio-Techne Tocris, Minneapolis, MN, USA).

### 2.15. Data Analysis

Data presented are means ± SEM. In some linear XY graphs, the data points are so similar that the symbols from individual points are superimposed. In some cases, the SEMs lie within the symbol. Scattered plot analysis was performed when appropriate. Means are compared by two-way ANOVA for comparison of more than two groups followed by Tukey’s or Sidak’s multiple comparisons tests per GraphPadPrism, v. 8.4.2., GraphPad Software, Inc., San Diego, CA, USA. A probability value less than 0.05 was considered statistically significant.

## 3. Results

### 3.1. Enzymes That Hydrolyze eATP Are Available on the Luminal Side of the Bladder Urothelium

**Figure 1:**
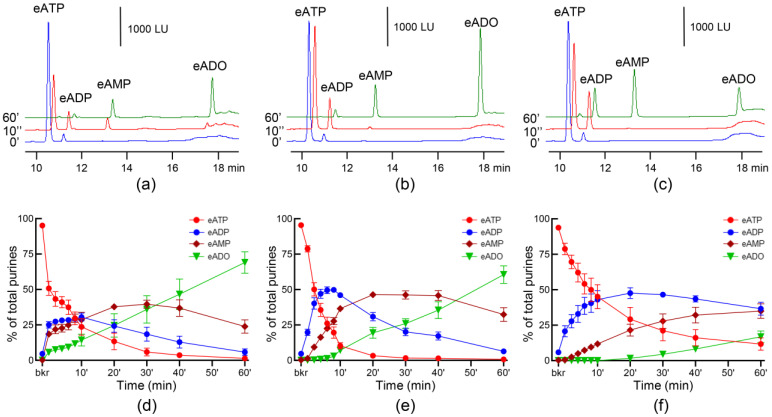
**Hydrolysis of eATP in bladder lumen of detrusor-free bladder preparations by membrane-bound and soluble nucleotidases.** Original chromatograms of eATP in beaker (blue, 0′, no enzymes present) and at 10 s (red) and 60 min (green) after starting the enzymatic reactions in the bladder lumen (**a**), in ILS collected at the end of bladder filling (**b**), and in ILS filtered through 10 kDa MWCO membranes (**c**). Note the decrease of eATP substrate and the increase or appearance of the e-products eADP, eAMP and eADO. Summarized data showing time-courses of the degradation of eATP in the bladder lumen, *n* = 4–6 (**d**), in ILS collected at the end of bladder filling, *n* = 6–14 (**e**), and in ILS collected at the end of bladder filling and filtered through 10 kDa MWCO membranes, *n* = 3 (**f**); *n*, number of bladders preparations. eATP, eADP, eAMP and eADO are presented as percentages of total purines (eATP + eADP + eAMP + eADO) present in reaction solutions at each time point for the duration of 1 h.

To determine whether ATP is degraded when instilled in the bladder lumen, detrusor-free bladder preparations were filled with KBS containing eATP, and the decrease in eATP and increase in eADP, eAMP, and eADO were evaluated in samples collected from the bladder lumen in the course of 1 h. 50% of eATP was degraded in the sample collected immediately after the bladder was filled to pre-voiding pressure (10 s of reaction) and was almost completely degraded after 30 min (Figure 1a,d). eADP was increased transiently in the first 10 min and reached approximately 25% of total purines, after which it declined gradually and was almost completely degraded at 60 min of reaction. eAMP and eADO raised gradually and at 60 min the aliquots of ILS contained ~1% eATP, ~4% eADP, ~20% eAMP, and ~75% eADO.

### 3.2. Enzymes That Hydrolyze eATP Are Released in the Bladder Lumen during Filling

To determine whether ATP-degrading enzymes were released in the bladder lumen during filling, eATP was added to the ILS that was removed from the bladder preparation at the end of filling. Then, substrate decrease and product increase were measured for 1 h following the addition of eATP. As shown in Figure 1b,e, eATP was quickly degraded to eADP, eAMP and eADO. In particular, eATP was decreased by ~20% in the first 10 s of reaction, the half-life of eATP (50% reduction) was approximately 2 min, ~90% of eATP was hydrolyzed within 10 min from start of reaction, and eATP was completely degraded by 20 min of reaction. Consequently, eADP increased transiently reaching 50% of total purines by 10 min of reaction. eAMP gradually increased and reached a plateau at 20 min of reaction, whereas eADO increased after 10 min and reached its highest proportion of total purines at the end of reaction. Thus, at 60 min the reaction solution contained ~0.5% eATP, ~4.5% eADP, ~30% eAMP, and ~65% eADO.

In ILS filtered by centrifugation through membranes with 10 kDa MWCO (Figure 1c,f), the degradation of eATP was delayed in comparison with the degradation of eATP in unfiltered ILS (Figure 1b,e). Thus, eATP was reduced by 50% at about 8 min of reaction and then gradually decreased; eADP and eAMP gradually increased and reached plateau at 10 min and 30 min, respectively; and, eADO was not formed in the first 30 min of reaction and then gradually increased. At 60 min, the reaction solution contained ~10% eATP, ~35% eADP, ~35% eAMP, and ~20% eADO.

### 3.3. Effects of Common ENTPDase Inhibitors on the Activities of s-NTDs in the Bladder Lumen

**Figure 2:**
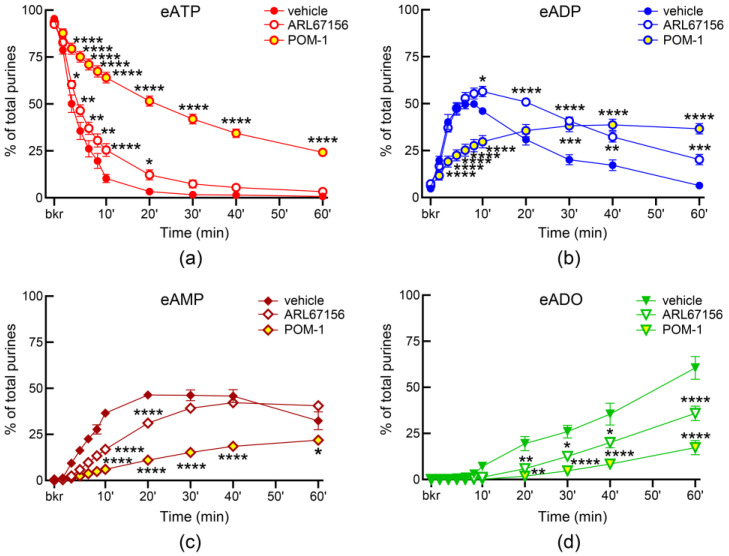
**Effects of ARL67156 and POM-1 on the hydrolysis of eATP by soluble enzymes released in bladder lumen during filling.** Changes in eATP (**a**), eADP (**b**), eAMP (**c**), and eADO (**d**) in ILS in the presence of vehicle (KBS, *n* = 6–14)) or ARL67156 (100 µM, *n* = 5) or POM-1 (100 µM, *n* = 3). eATP, eADP, eAMP and eADO are presented as percentages of total purines (eATP + eADP + eAMP + eADO) present in reaction solutions; *n*, number of bladders preparations. Asterisks denote significant differences from vehicle controls. * *p* < 0.05, ** *p* < 0.01, *** *p* < 0.001, **** *p* < 0.0001. 2 way ANOVA with Tukey’s multiple comparisons tests.

**Figure 3:**
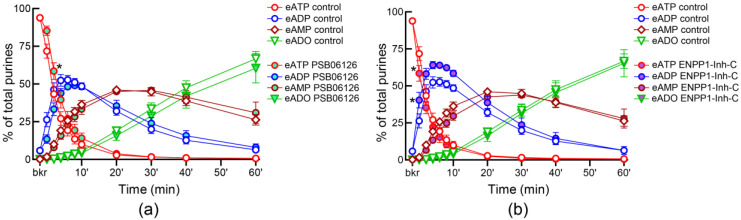
**Effects of PSB06126 and ENPP1-Inhibitor-C on the hydrolysis of eATP by soluble enzymes released in bladder lumen during filling.** Changes in eATP, eADP, eAMP, and eADO in ILS in the presence of vehicle (DMSO 0.1%, *n* = 7) or PSB06126 (10 µM, *n* = 3) (**a**) or ENPP-Inh-C (50 µM, *n* = 4) (**b**); *n*, number of bladder preparations. eATP, eADP, eAMP and eADO are presented as percentages of total purines (eATP + eADP + eAMP + eADO) present in reaction solutions. * *p* < 0.05 vs. control, 2 way ANOVA with Sidak’s multiple comparisons test.

**Figure 4:**
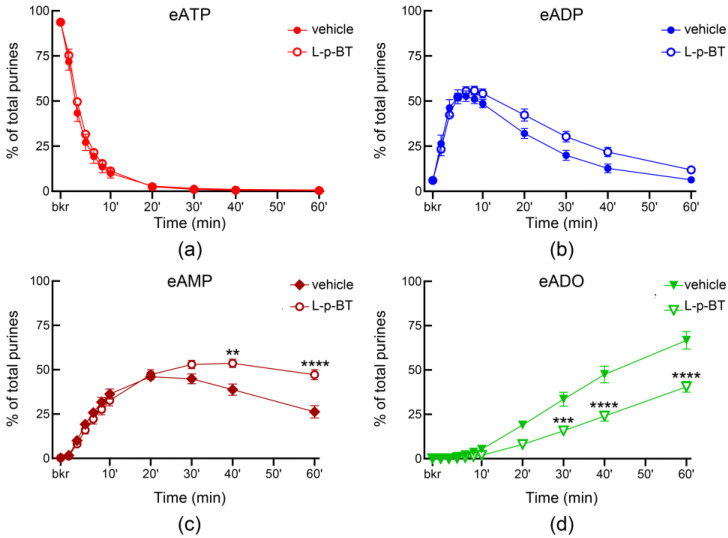
**Effect of L-*p*-BT on the hydrolysis of eATP by soluble enzymes released in bladder lumen during filling.** Changes in eATP (**a**), eADP (**b**), eAMP (**c**), and eADO (**d**) in ILS in the presence of vehicle (DMSO 0.1%, *n* = 7) or L-*p*-BT (100 µM, *n* = 3); *n*, number of bladder preparations. eATP, eADP, eAMP and eADO are presented as percentages of total purines (eATP + eADP + eAMP + eADO) present in reaction solutions. Asterisks denote significant difference from vehicle controls. ** *p* < 0.01, *** *p* < 0.001, **** *p* < 0.0001. 2 way ANOVA with Sidak’s multiple comparisons tests.

Both ARL67156 and POM-1, widely used ecto-nucleotidase inhibitors, inhibited the degradation of eATP in ILS so that the eATP decrease and the e-product increase were significantly diminished (Figure 2). POM-1 was a more potent inhibitor of the eATP hydrolysis than ARL67156. Note that the transient pattern of eADP increase (as seen in Figure 1d) was preserved in the presence of ARL67156. However, the eADP increase was sustained in the presence of POM-1 (Figure 2b). The ENTPD3 inhibitor PSB06126 tended to reduce the decrease in eATP at the beginning of reaction, but mostly it had no significant effect on the degradation of eATP (Figure 3a). ENPP1-Inhibitor-C tended to inhibit the degradation of eATP to eADP, but this effect reached statistical significance only at 10 s after addition of substrate to ILS (Figure 3b). L-*p*-BT (an inhibitor of ALPL/TNAP) had no effect on eATP decrease or eADP increase (Figure 4a,b). However, the formation of eADO was significantly reduced at 30–60 min of reaction while eAMP was accumulated at 40–60 min in the presence of L-*p*-BT (Figure 4c,d), suggesting that soluble enzyme(s) that degrade AMP to adenosine were likely inhibited by L-*p*-BT.

### 3.4. Enzymes That Hydrolyze AMP Are Released in the Bladder Lumen during Filling

#### 3.4.1. Degradation of eAMP in ILS from WT and Nt5e^−/−^ Bladders

**Figure 5:**
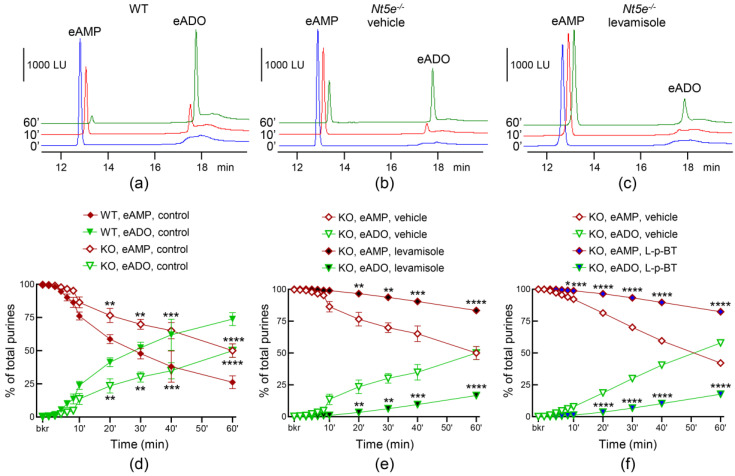
**Hydrolysis of eAMP by soluble nucleotidases in the lumen of bladder preparations from WT and *Nt5e^−/−^* mice.** Original chromatograms of eAMP in beaker (blue, 0′, no enzymes present), at 10 min (red), and at 60 min (green) after starting the enzymatic reactions in ILS collected at the end of bladder filling from WT controls (**a**), *Nt5e^−/−^* controls (**b**) and *Nt5e^−/−^* preparations treated with levamisole (1 mM) (**c**). Summarized data showing time-courses of eAMP decrease and eADO increase in ILS from WT bladders (*n* = 4–12) and from bladders isolated from *Nt5e^−/−^* mice (*n* = 4–7) (**d**), in *Nt5e^−/−^* preparations filled with either vehicle (KBS) or levamisole (*n* = 3) (**e**) or in *Nt5e^−/−^* bladders filled either with vehicle (DMSO 0.1%, *n* = 4) or L-*p*-BT (100 µM, *n* = 3) (**f**); *n*, number of bladder preparations. Note that the eAMP decrease and eADO increase were the greatest in WT preparations and that the eAMP hydrolysis was significantly inhibited in the presence of either levamisole or L-*p*-BT. ** *p* < 0.01, *** *p* < 0.001, **** *p* < 0.0001 vs. controls. 2 way ANOVA with Sidak’s multiple comparisons tests.

To determine whether NT5E/CD73 is released in the bladder lumen, we evaluated the degradation of eAMP in ILS from detrusor-free bladder preparations isolated from *Nt5e^−/−^* mice (Figure 5b,e) and compared it with the degradation of eAMP in ILS of WT (C57BL/6J) mice (Figure 5a,d). eAMP was degraded to eADO in ILS from both WT and *Nt5e^−/−^* preparations. In WT preparations, the degradation of eAMP was minimal in the first 6 min of reaction and at 10 min, about 25% of eAMP was degraded. Then, eAMP progressively declined while eADO progressively increased. The half-life of eAMP (e.g., 50% reduction) was ~30 min. In ILS from *Nt5e^−/−^* mice, the degradation of eAMP was slowed down significantly, so that only ~10% of eAMP was degraded in the first 10 min of reaction and 50% of eAMP was eliminated at 60 min. Therefore, it appeared that a soluble NT5E was released in the bladder lumen of WT bladder preparations during bladder filing.

#### 3.4.2. Effects of Levamisole and L-p-BT on the Degradation of eAMP in ILS from Nt5e^−/−^ Bladders

The degradation of eAMP in ILS from bladder preparations from *Nt5e^−/−^* mice was reduced but not abolished (as seen in Figure 5b,e). To determine whether an isoform of ALPL/TNAP was released in the bladder lumen and was responsible for the NT5E-resistant component of eAMP hydrolysis, we evaluated the degradation of eAMP in bladders isolated from *Nt5e^−/−^* mice in the presence of either levamisole (Figure 5c,e) or L-*p*-BT (Figure 5f), two inhibitors of ALPL/TNAP. Both inhibitors significantly inhibited the eAMP degradation in bladders lacking *Nt5e*.

### 3.5. Soluble Enzymes in the Bladder Lumen Are Similar to Known ENTPDases 

**Figure 6:**
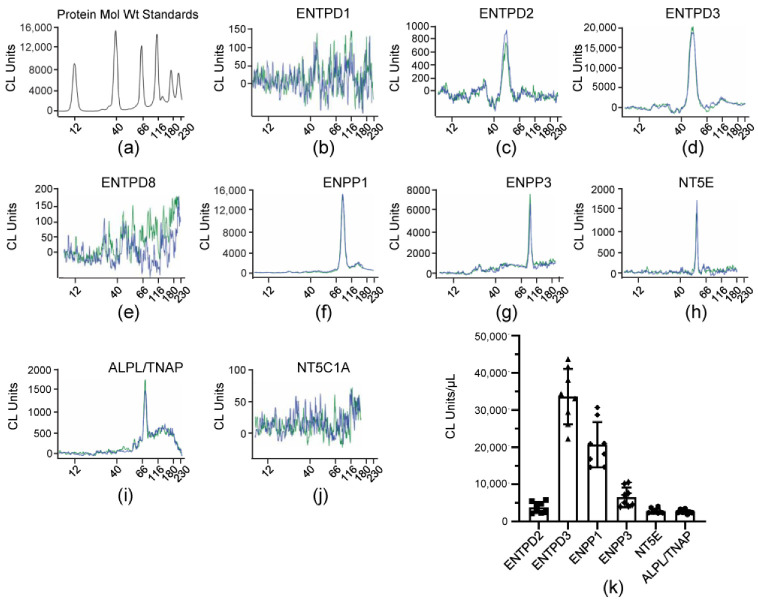
**Protein expression levels in ILS collected at the end of filling of detrusor-free bladder preparations.** (**a**–**j**) Representative immunoelectropherograms (duplicates) of nucleotidases evaluated using ProteinSimple Wes. Each antibody was diluted 100-fold and each well contained 3 µL of ILS sample. (**k**) Scatter plots of AUC of chemiluminescence (CL) signals normalized per µL loaded ILS sample (*n* = 8); *n*, number of bladder preparations. Statistical significance is described in Section 3.5.

ILS collected at the end of bladder filling of denuded bladder preparations were assessed by Wes analysis for expression of known membrane-bound nucleotidases that use ATP, ADP or AMP as substrates. The antibodies used for identification of ENTPD1, ENTPD2, ENTPD3, ENTPD8, ENPP1, ENPP3, and NT5C1A were validated in mouse brain homogenates or tissue homogenates in which the identical nucleotidase has not been detected per The Mouse Gene Expression Database and The Human Protein Atlas as shown previously [47]. In addition, each antibody was tested in mouse urothelium homogenates. Our initial findings with the ILS samples suggested that the antibodies we previously used to detect NT5E and TNAP [47] had reduced efficacy. Therefore, we used different NT5E and TNAP antibodies to detect NT5E and TNAP in ILS samples that were KO validated by the vendors. We re-probed urothelium homogenates with these antibodies. Slightly higher levels of NT5E and TNAP were found in urothelium homogenates, but these differences were not statistically significant (data not shown). The following nucleotidases were detected in ILS: ENTPD3 > ENPP1 >> ENPP3 ≥ ENTPD2 = NT5E = ALPL/TNAP (Figure 6k). ENTPD1 and ENTPD8 were detected in urothelium [47], but were not resolved in ILS (Figure 6b,e) whereas NT5C1A was not resolved in either urothelium or ILS (Figure 6k). ENTPD3 was the main nucleotidase detected in ILS (Figure 6d,k) (*p* < 0.0001 from all other nucleotidases detected in ILS) although it was the third highest protein expressed in the urothelium [47]. ENPP1 (Figure 6f,k) was the second highest nucleotidase in both urothelium homogenate [47] and ILS (*p* < 0.0001 from all other nucleotidases detected in ILS). The levels of ENPP3, ENTPD2, NT5E and ALPL/TNAP were significantly lower than ENTPD3 and ENPP1, but were not significantly different from each other (2 way ANOVA with Tukey’s multiple comparisons test).

### 3.6. Activation of Adenylyl Cyclases Stimulates the Release of s-NTDs in the Bladder Lumen and Inhibits the Release of s-NTDs in the Lamina Propria

**Figure 7:**
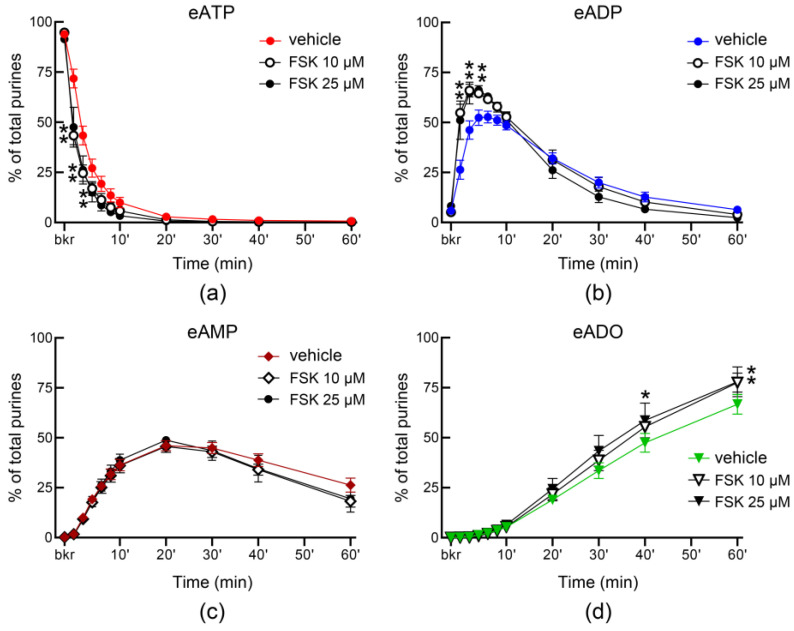
**Effect of forskolin (FSK) on the hydrolysis of eATP by soluble enzymes released in bladder lumen during filling.** Decrease of eATP (**a**) and increase in eADP (**b**), eAMP (**c**), and eADO (**d**) after addition of eATP in ILS from bladder preparations filled with vehicle (DMSO 0.1%, *n* = 7) or FSK (*n* = 4 each); *n*, number of bladder preparations. Note that the decrease of eATP and the formation of eADP and eADO were enhanced in the presence of FSK. Asterisks denote significant differences from vehicle controls. * *p* < 0.05, ** *p* < 0.01. 2 way ANOVA with Tukey’s multiple comparisons tests.

**Figure 8:**
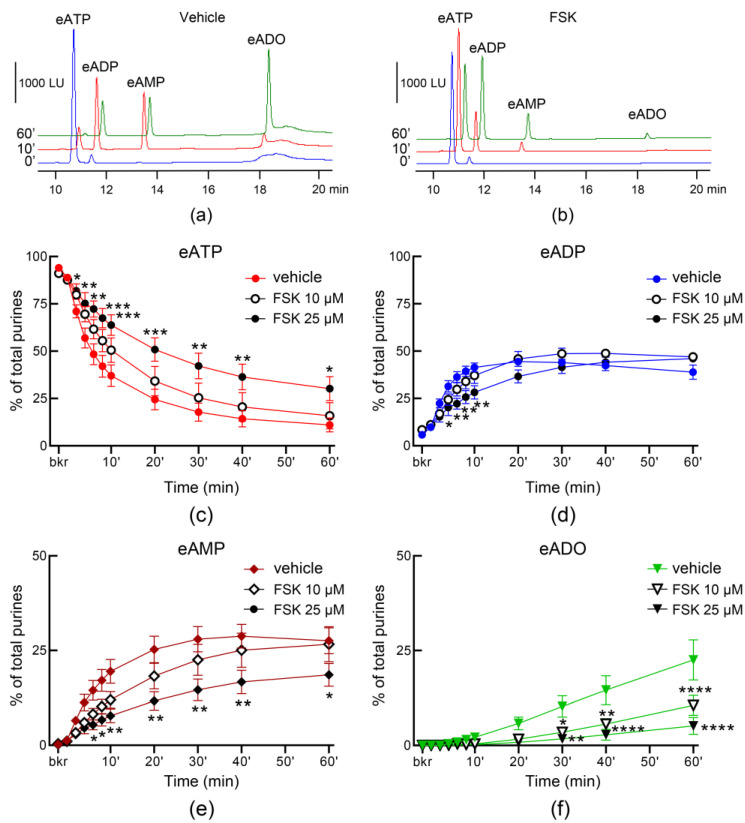
**Effect of FSK on the hydrolysis of eATP by soluble enzymes released in the bladder lamina propria.** Original chromatograms of eATP in beaker (blue, 0′), at 10 min (red), and at 60 min (green) after addition of eATP to concentrated ELS collected at the end of bladder filling in the presence of vehicle (DMSO 0.1%) (**a**) or FSK (25 µM) (**b**). Summarized data showing time-courses of eATP decrease (**c**), eADP increase (**d**), eAMP increase (**e**), and eADO increase (**f**) in the presence of vehicle (DMSO 0.1%, *n* = 7) or FSK (*n* = 4 each); *n*, number of bladder preparations. Note that the degradation of eATP was reduced in the presence of FSK. * *p* < 0.05, ** *p* < 0.01, *** *p* < 0.001, **** *p* < 0.0001 vs. vehicle controls. 2 way ANOVA with Tukey’s multiple comparisons tests.

A cAMP-protein kinase A-mediated pathway has been suggested to play a role in stretch-regulated exocytosis of discoid/fusiform vesicles (DVS) in bladder umbrella cells [7]. To determine whether activation of AC may regulate the distention-induced secretion of s-NTDs, we filled the bladder with a solution containing FSK and measured the hydrolysis of eATP in ILS collected from the bladder lumen at the end of filling. In the presence of 10 µM FSK, the decrease of eATP and increase of eADP were enhanced significantly in the first 4 min of reaction whereas eADO was significantly increased towards the end of reaction (Figure 7a,b,d), suggesting that higher amounts of s-NTDs were likely released in the bladder lumen during filling. Higher concentration of FSK (25 µM), did not result in greater s-NTDs release in the lumen (Figure 7). Previous studies have suggested that distinct mechanisms might regulate the release of s-NTDs on the opposite sides of the urothelium [4]. To determine whether activation of AC has similar effects on the release of s-NTDs in the lamina propria and in bladder lumen, we also evaluated the hydrolysis of eATP in concentrated ELS from detrusor-free preparations that were exposed to solutions containing FSK. As shown in Figure 8, FSK diminished the degradation of eATP in ELS collected from distended preparations in a concentration-dependent manner whereas no significant changes in the degradation of eATP in ELS collected from nondistended preparations were observed (data not shown).

### 3.7. Inhibition of Adenylyl Cyclases with SQ22536 Has Distinct Effects on the Release of s-NTDs in the Bladder Lumen and in Lamina Propria

**Figure 9:**
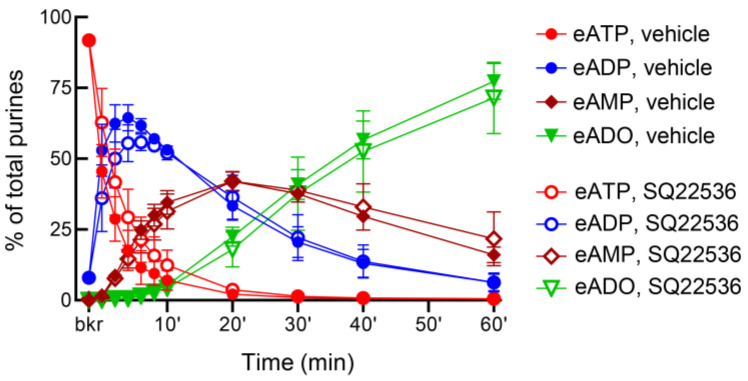
**Effect of SQ22536 on the hydrolysis of eATP by soluble nucleotidases released in the bladder lumen during filling.** The decrease in eATP and increase in eADP, eAMP, and eADO were not significantly different in the presence of vehicle (DMSO 0.2%, *n* = 4) or SQ22536 (100 µM, *n* = 3).

**Figure 10:**
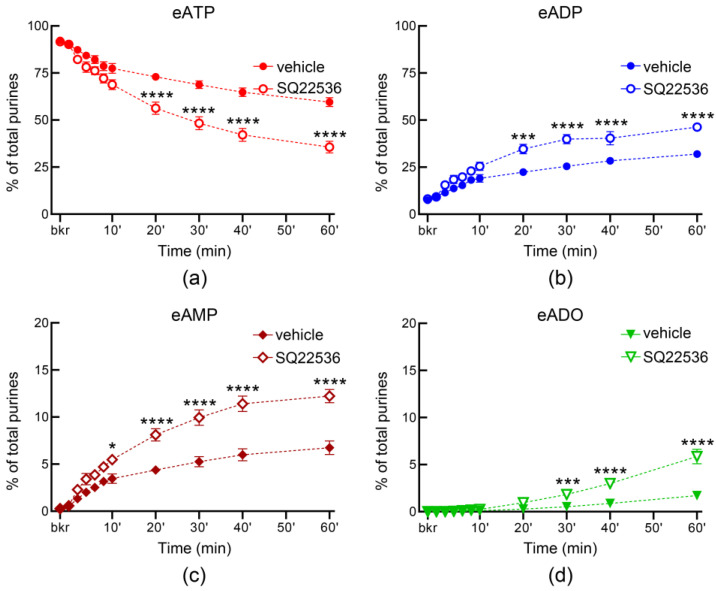
**Effect of SQ22536 on the hydrolysis of eATP by soluble nucleotidases released in the lamina propria of nondistended denuded bladder preparations.** Time courses of eATP decrease (**a**), eADP increase (**b**), eAMP increase (**c**), and eADO increase (**d**) after addition of eATP to concentrated ELS collected from nondistended (empty) preparations. Note that the degradation of eATP was enhanced in the presence of SQ22536 (100 µM, *n* = 3) in comparison to DMSO 0.2% control (*n* = 4); *n*, number of bladder preparations. * *p* < 0.05, *** *p* < 0.001, **** *p* < 0.0001 vs. vehicle controls. 2 way ANOVA with Sidak’s multiple comparisons tests.

**Figure 11:**
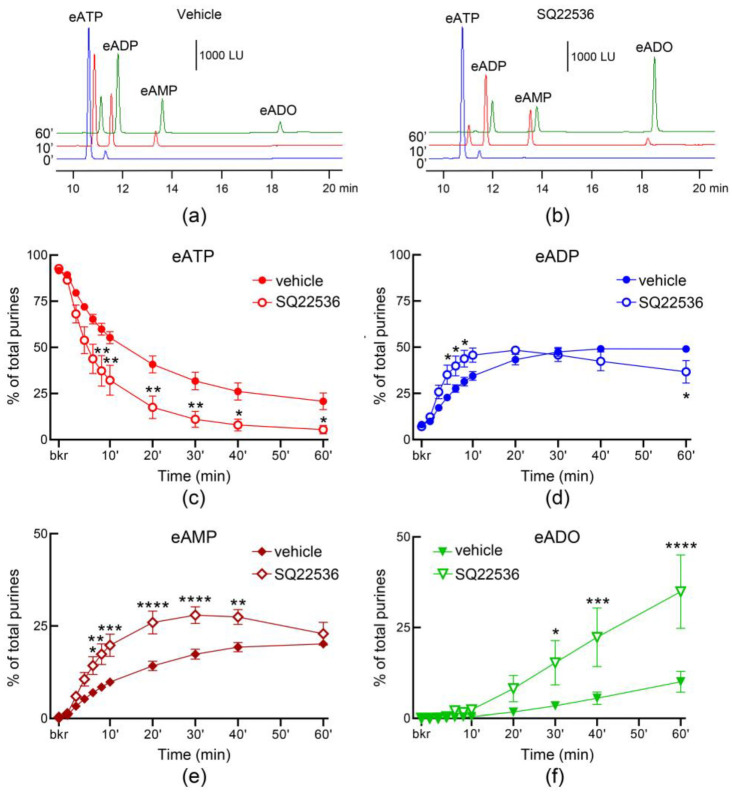
**Effect of SQ22536 on the hydrolysis of eATP by soluble nucleotidases released in the lamina propria of distended denuded bladder preparations**. Original chromatograms of eATP in beaker (blue, 0′), at 10 min (red), and at 60 min (green) after addition of eATP to concentrated ELS collected at the end of bladder filling in the presence of vehicle (DMSO 0.2%) (**a**) or SQ22536 (100 µM) (**b**). Summarized data of time courses of eATP decrease (**c**), eADP increase (**d**), eAMP increase (**e**), and eADO increase (**f**) after addition of eATP to concentrated ELS collected at the end of bladder filling. Note that the degradation of eATP was enhanced in the presence of SQ22536 (*n* = 3) in comparison with DMSO 0.2% control (*n* = 4). * *p* < 0.05, ** *p* < 0.01, *** *p* < 0.001, **** *p* < 0.0001. 2 way ANOVA with Sidak’s multiple comparisons tests.

In ILS, the AC inhibitor SQ22536 (100 µM) had no effect on the release of s-NTDs when compared with the vehicle (Figure 9). However, SQ22536 increased the release of s-NTDs in ELS collected from nondistended (Figure 10) and from distended (Figure 11) bladder preparations. Acceleration of eATP hydrolysis occurred at all sequential steps of enzymatic degradation of eATP to eADP and eAMP and then to eADO in the presence of SQ22536.

### 3.8. Adenosine Facilitates the Release of s-NTDs in the Bladder Lumen and Lamina Propria

**Figure 12:**
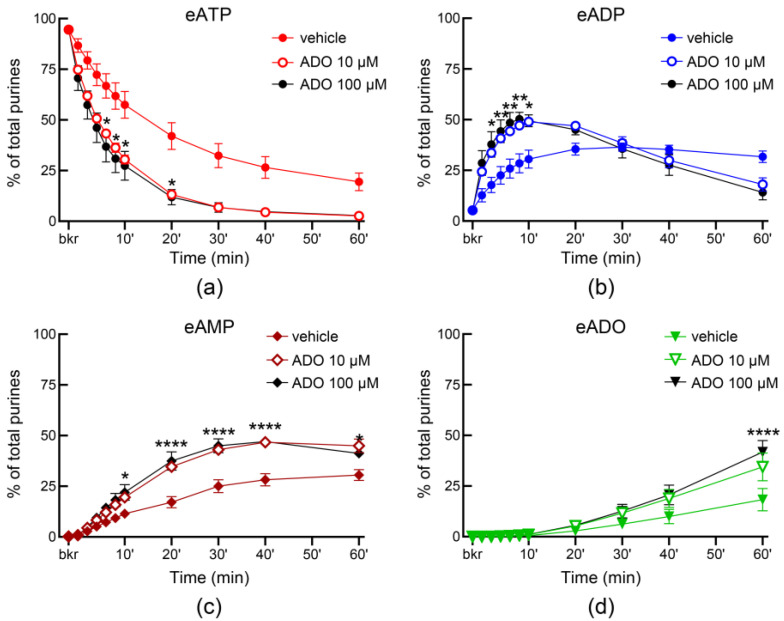
**Effects of adenosine (ADO) on the release of soluble nucleotidases in the bladder lumen.** Time courses of eATP decrease (**a**), eADP increase (**b**), eAMP increase (**c**), and eADO increase (**d**) after addition of eATP to concentrated ILS collected at the end of bladder filling with either vehicle (KBS, *n* = 10) or ADO (*n* = 4); *n*, number of bladder preparations. Note that the degradation of eATP was enhanced in ILS from bladders filled with ADO. * *p* < 0.05, ** *p* < 0.01, **** *p* < 0.0001 vs. vehicle control. 2 way ANOVA with Sidak’s multiple comparisons tests.

**Figure 13:**
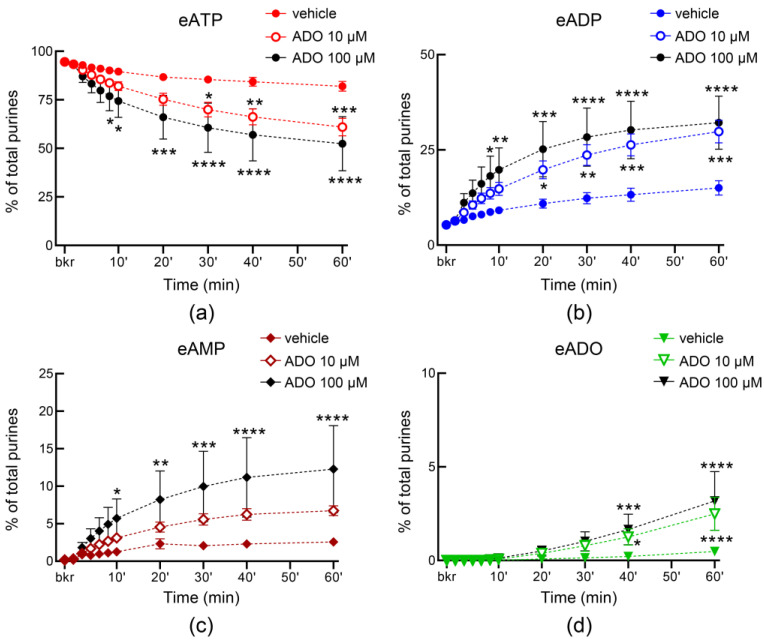
**Effects of adenosine (ADO) on the release of soluble nucleotidases in lamina propria of nondistended denuded bladder preparations.** Time courses of eATP decrease (**a**), eADP increase (**b**), eAMP increase (**c**), and eADO increase (**d**) after addition of eATP to ELS collected from nondistended bladder preparations treated with either vehicle (KBS, *n* = 9) or ADO (*n* = 4 each) for a time period equivalent to the time for bladder filling; *n*, number of bladder preparations. Note that the degradation of eATP was enhanced in concentrated ELS collected from preparations treated with ADO. * *p* < 0.05, ** *p* < 0.01, *** *p* < 0.001, **** *p* < 0.0001 vs. vehicle controls. 2 way ANOVA with Tukey’s multiple comparisons tests.

**Figure 14:**
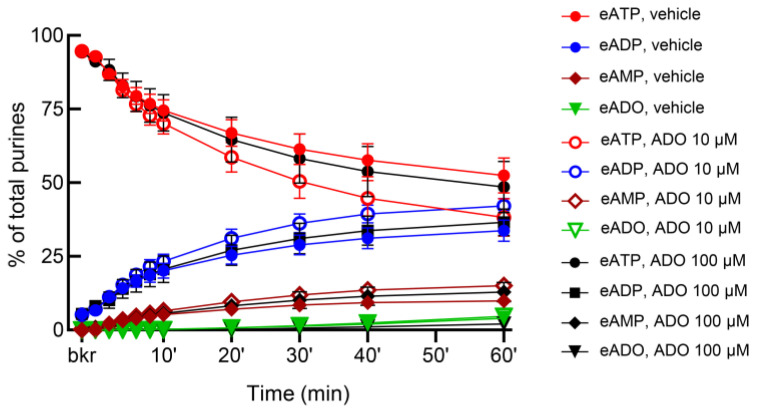
**Effects of adenosine (ADO) on the release of soluble nucleotidases in lamina propria of distended denuded bladder preparations.** eATP decrease and eADP, eAMP, and eADO increase after addition of eATP to concentrated ELS collected at the end of bladder filling. Note that there is no significant difference between eATP degradation in ELS from bladder preparations treated with vehicle (KBS, *n* = 9) and adenosine (*n* = 4 each); *n*, number of bladder preparations.

Adenosine has been suggested to be released from the bladder mucosa in response to hydrostatic pressure and to stimulate umbrella cell exocytosis [58]. Moreover, adenosine is available in lamina propria and bladder lumen during filling [4]. Therefore, we next tested whether the release of s-NTDs in ILS or ELS is altered by adenosine. As shown in Figure 12, adenosine (10 µM and 100 µM) enhanced the release of s-NTDs in ILS and consequently it accelerated the decrease of eATP and the increase of eADP, eAMP, and eADO. There was no difference between the effects of the two concentrations of adenosine in ILS, suggesting that maximum effect of adenosine was likely reached at 10 µM. The release of s-NTDs in concentrated ELS collected from nondistended preparations was also enhanced in the presence of adenosine (Figure 13). Thus, the substrate decrease and product increase were greater in the presence of adenosine than in vehicle controls. However, the release of s-NTDs in ELS collected at the end of filling (i.e., distended preparations) remained untouched in the presence of adenosine (Figure 14).

## 4. Discussion

Distention-induced release of ATP from urothelial cells mediates excitation of sensory neurons in the urothelium and lamina propria to evoke voiding reflex [59], and it has been proposed that voiding dysfunctions associated with LUT or systemic diseases may be related to augmented ATP release from the urothelium [5,60]. Moreover, intravesical instillation of ATP has been found to increase bladder activity, which was interpreted as indication that not only ATP in the suburothelial layer, but also ATP in the bladder lumen, are involved in initiation of voiding [61,62]. Possible involvement in bladder excitability disorders makes it important to understand mechanisms by which effective concentrations of ATP at the vicinity of P2 purinergic receptors in the bladder wall and in the urine are regulated. In the present study, we investigated the hydrolysis of ATP by soluble nucleotidases that were released in the bladder lumen during filling. We found that (1) s-NTDs are released in the bladder lumen during bladder filling in a regulated manner, and (2) there is differential control of release of s-NTDs at the luminal/mucosal and abluminal/lamina propria surfaces of the urothelium during filling.

To eliminate the potential influence of sources of s-NTDs outside of the bladder such as plasma filtered in the kidneys or upper urinary tract epithelium, we carried out the study in a decentralized (ex vivo) murine bladder model with intact bladder mucosa but no detrusor [4]. The model allowed us to investigate local mechanisms in the bladder uroepithelium, to expose the bladder mucosa to physiologically relevant distention as the bladder preparation is filled, and to obtain direct access to both surfaces of the urothelium during filling. We previously reported techniques to evaluate the hydrolysis of adenine nucleotides using highly fluorescent substrates (i.e., eATP) and ultra-sensitive liquid chromatography [63,64,65]. Using these approaches, we recently discovered mechanosensitive release of s-NTDs in the lamina propria of the murine bladder [47]. In the present study, we studied the hydrolysis of eATP to eADP, eAMP, and eADO in intraluminal solutions collected at the end of bladder filling and found that highly efficient s-NTDs were released in the bladder lumen. A direct comparison between activities of e-NTDs and s-NTDs in the bladder lumen was difficult to draw because the two types of activity (i.e., e-NTDs and s-NTDs) could not be separated by introducing the substrate in the bladder lumen. Instead, filling of the bladder with solution containing the substrate eATP provided information about the “combined” activities of e-NTDs and s- NTDs in the bladder mucosa. On the other hand, carrying out reactions in solutions that were removed from the bladder prior to addition of substrate, enabled us to isolate the s-NTDs activities in the bladder lumen. The patterns of eATP degradation in the presence and absence of tissue were remarkably similar. Thus, in both cases eATP steeply decreased, eADP transiently increased, and eAMP and eADO gradually increased, the latter product reaching its peak towards the end of reaction (Figure 1). These data clearly demonstrate that highly efficient s-NTDs that sequentially degrade ATP to ADP, AMP and adenosine were released in the bladder lumen during bladder filling. These findings have at least two important implications: (1) urinary ATP is regulated by its hydrolysis in the bladder lumen, and (2) intraluminal s-NTDs might be novel targets for rectification of aberrant ATP concentrations in the bladder lumen during filling.

Previous studies have suggested that ATP might be degraded at the luminal surface of the bladder mucosa. Thus, bladder umbrella cells appear to express ATP-metabolizing enzymes [66,67] and ATP metabolites have been found in the lumen of mouse and monkey bladders [3,4] and in human urine samples [23,68]. In contrast, studies in rabbit mucosa sheets mounted in Ussing chambers failed to detect hydrolysis of exogenous ATP added to the mucosal chamber [8,69]. In these studies, however, the concentrations of endogenous ATP appeared to decline gradually in the mucosal chamber in response to increase of hydrostatic pressure [8]. It is possible that at least part of the ATP decline was due to ATP degradation, but levels of ATP metabolites were not measured in these studies.

Here, we provide direct evidence that ATP was degraded to ADP, AMP, and adenosine in the bladder lumen by both e-NTDs present in the bladder mucosa and s-NTDs released from the urothelium during bladder filling. The presence of all products of the sequential ATP hydrolysis in ILS suggests that the s-NTD pool in the bladder lumen consists of multiple enzymes. Centrifugation of ILS through membranes with 10 kDa MWCO resulted in modified patterns of substrate decrease and product increase so that the transient increase of eADP in unfiltered ILS was replaced with a gradual and slow increase of eADP in filtered ILS. Likewise, the formation of eAMP and eADO was delayed and diminished in filtered ILS in comparison with unfiltered ILS. These changes suggested that s-NTDs with various sizes (<10 kDa and >10 kDa) were released in the bladder lumen during filling. Distinct patterns of eATP degradation were found in the bladder lumen (the present study) and in lamina propria/ELS [47] processed in identical manners, suggesting that either the types or the relative amounts of s-NTDs released on both sides of the urothelium differ.

As part of general characterization of s-NTDs released in the bladder lumen, we tested the effects of common nucleotidase inhibitors on eATP hydrolysis. The non-specific ENTPDase inhibitors POM-1 and ARL67156 diminished the eATP degradation, with POM-1 causing greater enzyme inhibition than ARL67156. Using the highly sensitive and specific Wes assay (ref. [70,71]), we determined that ENTPD3 and ENPP1 were the dominant s-NTDs among the detected NTDs in ILS. However, PSB06126 and ENPP1 Inhibitor C did not inhibit the degradation of eATP in a substantial way, suggesting that enzyme inhibitors may not always have sufficient specificity and/or efficacy to determine the identity of released enzymes. A previous study in the same bladder model demonstrated that the mouse urothelium expresses relatively high amounts of ENTPD1 [47]. However, this protein was not resolved in the ILS assayed in the present study. Other differences between relative expression of NTDs in ILS and urothelium were also observed. Thus, ILS contained ENTPD3 > ENPP1 > ENPP3 ≥ ENTPD2 = NT5E = ALPL/TNAP whereas the mouse urothelium had ENTPD1 >> ENPP1 > ENTPD2 = ENTPD3 > ENPP3 = NT5E >> ENTPD8 = TNAP [47]. Such differences suggested that release of s-NTDs in the bladder lumen during filling did not result from cell or tissue damage. The relative distribution of enzymes in ILS was also different from the s-NTDs distribution in ELS; the latter samples contained ENTPD1 >> ENTPD3 >> ENPP3 > ENPP1 = ENTPD2 = NT5E >> ENTPD8 = TNAP [47]. Together, these findings suggest that regulated release of s-NTDs in bladder lumen and in lamina propria occur during bladder filling. Soluble forms of nucleotidases have been reported for blood plasma, cultured astrocytes, endothelial cells, and sympathetic nerve terminals [49,50,72]. Membrane proteins, including single-pass or dual-pass transmembrane proteins, can be released via proteolytic ectodomain shedding [73] or via membrane microparticles, microvesicles or exosomes [49,74]. Further studies are warranted to determine how s-NTDs are released from the bladder urothelium.

Urothelium and suburothelium/lamina propria are the two layers of the detrusor-free mouse bladder preparation used in the present study [4]. The results obtained with Wes methodology for protein analysis in the present study showed similarities and differences with previous immunohistochemistry studies in mouse bladder [45,46]. For example, both studies demonstrated the presence of ENTPD1, ENTPD2, ENTPD3 and ALPL in urothelium/lamina propria whereas ENTPD8 had very low expression, if any. In the immunohistochemistry studies, ENTPD1, ENTPD2 and ALPL appeared to be localized in cells in the lamina propria but not in the urothelium whereas ENTPD3 was found in basal and intermediate cells of the urothelium but not in lamina propria, and ENPP1 and ENPP3 were not investigated [45,46]. None of the enzymes that were investigated were detected in the apical membranes of umbrella cells [45,46]. However, other studies have reported that bladder umbrella cells are a place for ATP and ADP hydrolysis [66] and ENTPD3 localization [67]. No NT5E was detected in the mouse bladder urothelium and lamina propria in the study of [45], whereas we determined that NT5E is present in the mouse urothelium and is released in the bladder lumen during bladder filling (discussed below). These discrepancies between studies are likely due to the use of different methodologies and tools (e.g., antibodies) for protein analysis.

NT5E/CD73 is the major enzyme that dephosphorylates AMP to generate extracellular adenosine [75,76], but this reaction can also be carried out by ALPL/TNAP [77]. Moreover, it has been suggested that the expression of ALPL/TNAP can be increased in the absence of functional NT5E/CD73 [78]. To investigate whether NT5E/CD73 was released in the bladder lumen during filling, we evaluated the hydrolysis of eAMP in ILS collected from bladder preparations isolated from *Nt5e^−/−^* mice. The degradation of eAMP and formation of eADO was significantly diminished (but not abolished) in bladder preparations with completely deleted *Nt5e* gene, suggesting that NT5E/CD73 was released in the ILS of WT bladders during filling. To determine whether ALPL/TNAP was responsible for the remaining component of the eAMP degradation in *Nt5e^−/−^* preparations, we measured the eAMP conversion to eADO in *Nt5e^−/−^* bladders filled with KBS containing either levamisole or L-p-BT, common inhibitors of ALPL/TNAP [79]. Both inhibitors almost abolished the remaining component of eAMP hydrolysis, suggesting that ALPL/TNAP was likely released in the bladder lumen during filling. These functional studies were supported by detection of NT5E/CD73 and ALPL/TNAP in ILS by ProteinSimple Wes. NT5E/CD73 and ALPL/TNAP are both attached to the plasma membrane by a glycosylphosphatidylinositol (GPI) anchor and are found in plasma, possibly as a consequence of cleavage of GPI by GPI-specific phospholipase activity [49,73]. Similar mechanisms might underlie the release of these enzymes in the bladder lumen in response to bladder wall distention.

Next, we asked whether the release of s-NTDs in the bladder lumen during filling is a regulated process. Bladder urothelium umbrella cells contain a large pool of subapical discoidal or fusiform vesicles (DFVs) [80,81] that undergo regulated exocytosis in response to stretch [82,83]. Thus, the apical cell membranes of the umbrella cells adapt to increasing tension by increasing surface with DFVs exocytosis. DFVs are also transport carriers for cargo molecules such as uroplakins, human growth hormone, proteases and perhaps other proteins that are delivered to the apical surface of the umbrella cells allowing for vesicle exocytosis [83]. It is proposed that stretch stimulates AC and production of cyclic AMP, which in turn activates exocytosis of DFVs [7,84]. If release of s-NTDs in the bladder lumen is associated with these processes, one would expect stimulation of AC to increase release of s-NTDs and potentiate the ATP hydrolysis in ILS. The activator of AC FSK enhanced briefly, however significantly, the degradation of eATP to eADP and tended to increase eADO formation although this effect did not reach statistical significance. Increasing the concentration of FSK did not enhance further the s-NTD release in the lumen, suggesting that maximum activation of AC in umbrella cells was likely achieved with the lower dose of FSK. The AC inhibitor SQ22156 had no effect on the release of s-NTDs in the lumen. It appears therefore, that the AC-cyclic AMP pathway might contribute to the regulation of s-NTD release in umbrella cells, but this mechanism may not play a dominant role. Alternatively, it might be that the exposure of the urothelium to FSK only during the filling phase was insufficient to cause measurable changes in DVS exocytosis, because a previous study reported changes in umbrella cell morphology after treating uroepithelium sheets for several hours with FSK and a phosphodiesterase inhibitor simultaneously [7]. Finally, it might be that the mouse umbrella cells express AC isoforms with low sensitivity to FSK. For example, FSK appears to be a weak activator of AC9 [85]. In contrast to the situation in the bladder lumen, FSK dose-dependently diminished the distention-induced release of s-NTDs without altering the constitutive release of s-NTDs. In contrast, the AC inhibitor SQ22156 enhanced both the constitutive and the distention-induced release of s-NTDs in the lamina propria. These are intriguing observations, suggesting that (1) there is a basal activity of AC in the lamina propria that suppresses spontaneous s-NTDs release, (2) distention of the bladder wall further activates AC in lamina propria, and (3) different AC-mediated mechanisms regulate the release of s-NTDs at the luminal and abluminal sides of the urothelium.

The apical plasma membrane of umbrella cells can also be modulated by external stimuli, independent of stretch. For example, adenosine has been proposed as an autocrine/paracrine modulator of apical exocytosis [58] that is mediated by the A1 adenosine receptor/phospholipase C/protein kinase C/ADAM17 signaling pathway [86]. Release of adenosine from the bladder mucosa during filling or stretch has been demonstrated [87]. In the present study, adenosine enhanced the release of s-NTD in the bladder lumen during filling and consequently accelerated the hydrolysis of ATP. Likewise, adenosine increased the constitutive, but not the distention-induced, release of s-NTDs in the lamina propria, suggesting that its effects in the lamina propria may be independent of stretch. To our knowledge, adenosine-induced ATP hydrolysis has not been demonstrated previously. The role of this mechanism in regulation of bladder excitability remains to be determined. In a study of rabbit bladder mucosa mounted in Ussing chamber, ATP levels in the serosal chamber were reduced in the presence of adenosine. This effect was attributed to A1 mediated inhibition of distention-induced ATP *release* [88]. The possibility that adenosine might have accelerated the metabolism of released ATP resulting in reduced levels of ATP was not considered.

ATP and adenosine are frequently described as causing opposite effects on functions of excitable cells. Thus, by stimulating release of s-NTDs, adenosine might be promoting its own production to lessen actions of too much extracellular ATP. As discussed above, at the end of filling, adenosine is the dominant purine substance at both surfaces of the urothelium while ATP comprises only 5–10% of total purines [3,4]. Such low amounts of ATP at the end of filling cannot easily explain its assumed role as the primary mediator from the urothelium that activates the micturition reflex at end of filling [59]. Along these lines, a study in humans reported greater intraluminal ATP concentrations at early filling stages than at later filling stages in women with and without detrusor overactivity [11]. A recent study that performed micturition analysis, video urodynamics, and urothelial ATP release measurements in control and transgenic mice, concluded that ATP is *not* essential for the normal micturition reflex [60]. On the other hand, it has been proposed that adenosine, acting through A1 adenosine receptors, stimulates bladder function by lowering the threshold for micturition [87] or by enhancing bladder afferent stimulation [89]. Therefore, enhanced formation of adenosine through adenosine-induced ATP hydrolysis would be compatible with the idea that adenosine, but not ATP, is a key signal from the urothelium that initiates voiding at the end of bladder filling. Therefore, evidence provided in the present study suggests that such possibilities as well as long held theories should be further explored.

Despite controversies about the physiological role of ATP in bladder functions, there seems to be a consensus that high levels of ATP in the bladder lumen are associated with increased bladder excitability and with voiding dysfunctions [59,60,90,91]. How is ATP increased in the bladder lumen or in lamina propria is of utmost importance because knowing the mechanisms underlying ATP increase would focus the efforts to normalize ATP levels at the correct target. At present, almost all studies that detect increased levels of ATP in the bladder lumen presume increased *release* of ATP in the extracellular space with no direct evidence. Given the broad range of possible ATP release mechanisms, targeting ATP release might be a daunting task especially if the cause of ATP increase is different from aberrant ATP release mechanisms. Pharmacological targeting of purinergic receptors selectively has also been challenging due to the ubiquitous distribution and expression of the receptors, distinct effects on different cell types, variable levels of the endogenous agonists, receptor desensitization over time, and redundancy of purinergic signaling [92]. It has been recognized that the increase of ATP may result not only from an excessive release of ATP, but also from reduced extracellular hydrolysis of the molecule by ecto-nucleotidases [22,93]. Accordingly, acceleration of extracellular ATP metabolism by ecto-nucleotidases has been suggested as a potential treatment strategy for overactive bladder or interstitial cystitis and bladder pain syndrome [94]. Targeting membrane-bound nucleotidases, however, could also be challenging due to their ubiquitous distribution and expression and their incorporation in the plasma membrane. In this regard, targeting ATP hydrolysis by bladder-specific s-NTDs could be more efficacious. In light of the findings in the present study, it cannot be excluded that inhibited hydrolysis of released ATP could be the basis of elevated urinary ATP that is considered a cause of voiding dysfunctions associated with LUT and systemic diseases. Conversely, it can be speculated that low levels of urinary ATP in patients with UAB [20] may be caused by accelerated degradation of ATP in the bladder lumen due to increased release of s-NTDs. Although the value of urinary ATP as a clinical surrogate test for certain urinary tract pathologies has been questioned in some studies [95], the relevance of urinary ATP in bladder physiology and pathophysiology is still very evident and merits further studies.

In summary, we have demonstrated release of s-NTDs in the bladder lumen during filling of urinary bladders of adult mice, which can control the amount of ATP in the bladder lumen, and have determined the identity of several s-NTDs. Therefore, the extracellular concentrations of ATP in the bladder lumen and in lamina propria are determined not only by its spontaneous and stretch-induced release, but also by its degradation by both membrane-bound and soluble enzymes. Moreover, we have uncovered signaling pathways that regulate the release of s-NTDs in the bladder lumen and lamina propria. These discoveries broaden our understanding of purinergic regulation of bladder excitability.

## Data Availability

The raw data supporting the conclusion of this article will be made available by the authors upon reasonable request. The data are not publicly available due to privacy.
